# Supplementary material for: Experimental quantification of hydrogen content in the Earth’s core
Source: Nat Commun. 2026 Feb 10;17:1211. doi: 10.1038/s41467-026-68821-6 (PMC12890951; doi:10.1038/s41467-026-68821-6)
Supplement: Supplementary file 1 — Supplementary Information [file 41467_2026_68821_MOESM1_ESM.pdf]

Supplementary Materials for Experimental quantification of hydrogen content in the Earth's core

Dongyang Huang*, Motohiko Murakami, Stephan Gerstl, Christian Liebske
*Corresponding author. Email: dhuang@pku.edu.cn

This PDF file includes:

Figures S1 to S5

Other Supplementary Materials for this manuscript:

Supplementary Data 1 to 7.
APT data is available through https://doi.org/10.17632/8x4h7jt393.1, which contains the atom probe data, viz. mass spectrum, pos file and range file, for the metals (Figs. 2–3) and silicates (Figs. S3–S4).

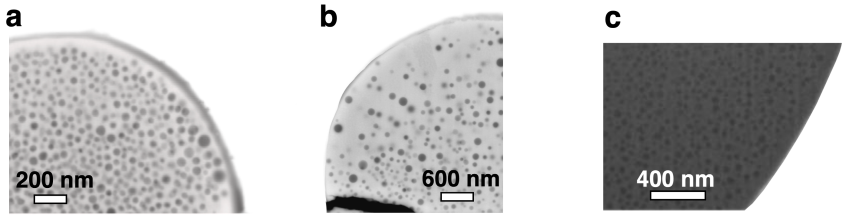

Fig. S1 Si–O-rich nanostructure formed within liquid iron-rich alloys during quenching in laser-heated diamond anvil cell experiments. (**a**–**c**) Backscattered electron images from the literature [1–9], showing the Si–O-rich quench texture (darker 'bubbles', typically < 200 nm) embedded within liquid iron-rich alloys (brighter matrix). These alloys previously equilibrated with molten silicates, following the partitioning of Si and O from silicate melts (see the main text). This repeatedly observed nanostructure has now been sampled and analysed, for the presence of H, using atom probe tomography, as reported in Figs. 1–3.

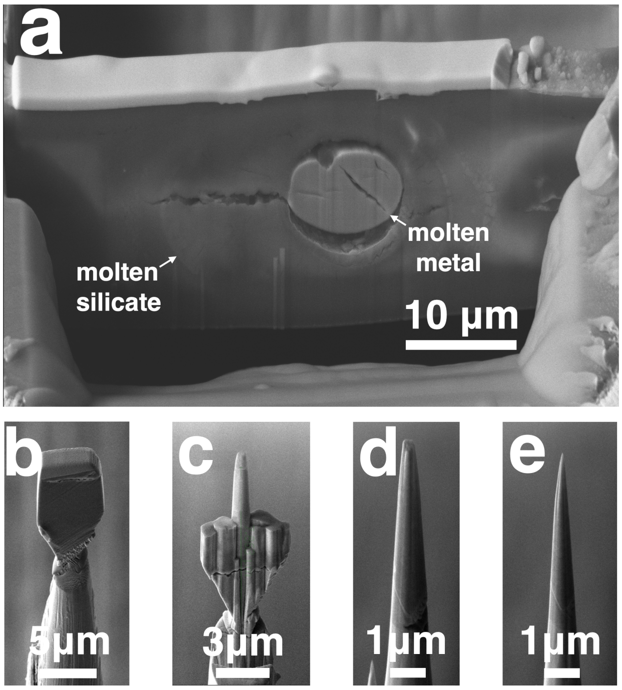

Fig. S2 Atom probe specimen preparation for laser-heated diamond anvil cell experiment. (**a**) Backscattered electron image showing the high-pressure equilibrated metal–silicate pocket (region-of-interest), obtained from standard FIB milling procedure designed for superliquidus experiments using laser-heated diamond anvil cell [5]. (**b**–**e**) Sharpening of the sample, be it metal or silicate, using low-current circular milling [10] until reaching the nanoscale region-of-interest and the diameter of <100 nm.

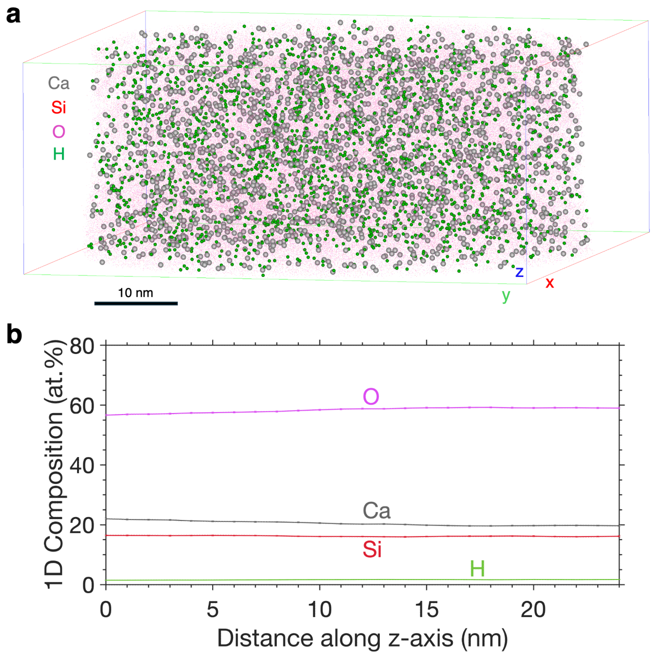

Fig. S3 APT analysis of Ca silicate perovskite (i.e. davemaoite) in the molten silicate pocket in equilibrium with the metal in Fig. 2. (**a**) Atom map of davemaoite, obtained from close to the edge of the molten silicate pocket. Due to very high failure rate, although many specimens were prepared from the same metal–silicate pocket (Fig. S2), we only obtained sufficient ion counts ($>$ 1 million) for davemaoite. This is in agreement with previous melting experiments, where davemaoite is the liquidus phase of the basaltic composition [11, 12]. H, labelled as large green spheres in **a**, shows homogeneous distribution within davemaoite, implying its artificial origin. (**b**) 1D composition profile along the z-axis, indicating the nearly stoichiometric $CaSiO_3$ composition throughout the sample apex. The invariably constant H content, 1.3 at.% (Supplementary Data 4), is however indistinguishable from the residual H (see the main text).

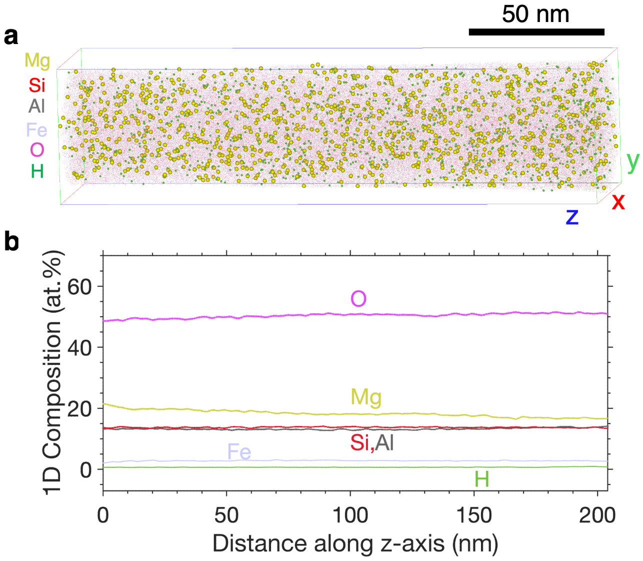

Fig. S4 APT analysis of the molten silicate in equilibrium with the metal in Fig. 3. (**a**) Atom map of the molten silicate, consisted of evenly distributed major rock-forming elements, viz. Mg, Si, Al, Fe and O. Low concentrations of Ca (0.5 at.%, Supplementary Data 5) is not shown here, and is in line with the observation that Ca silicate perovskite crystallises first during the cooling of the basaltic melt (cf. Fig. S3). (**b**) 1D composition profile along the z-axis. Again, the ∼0.5 at.% H may be entirely stemming from the residual H.

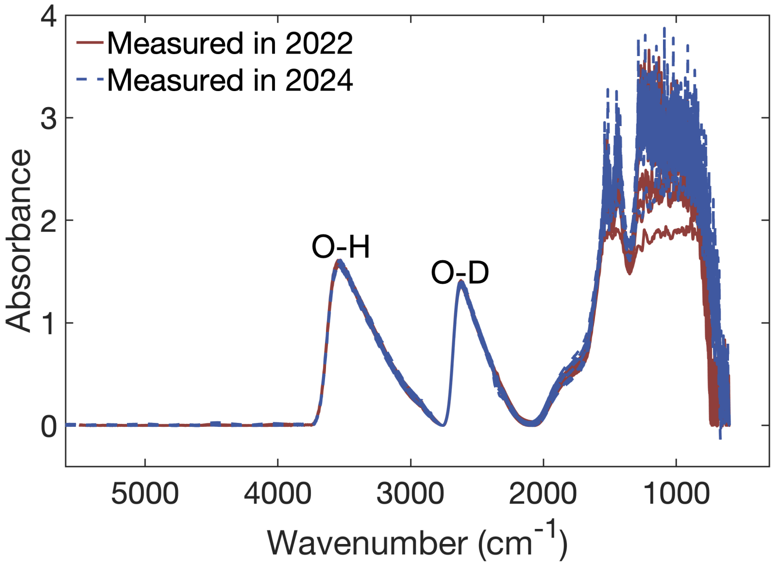

Fig. S5 Infrared spectrum from the starting hydrous basaltic glass. Prominent OH and OD absorbance peaks are observed at ∼3500 cm^{-1} and ∼2600cm^{-1}, respectively. The measurements were performed twice, under the same FTIR configurations, in a time interval of two years, during which the sample was kept in a lab drawer. The estimated water contents are (i) 6467 ±56 ppm H_2O and 7553 ± 98 D_2O, respectively (details see [13]), and (ii) identical from the year of 2022 and 2024.

References

[1] J. Siebert, J. Badro, D. Antonangeli, F.J. Ryerson, Metal-silicate partitioning of Ni and Co in a deep magma ocean. Earth and Planetary Science Letters **321-322**, 189–197 (2012). https://doi.org/10.1016/j.epsl.2012.01.013. URL http://dx.doi.org/10.1016/j.epsl.2012.01.013

[2] R.A. Fischer, Y. Nakajima, A.J. Campbell, D.J. Frost, D. Harries, F. Langenhorst, N. Miyajima, K. Pollok, D.C. Rubie, High pressure metal-silicate partitioning of Ni, Co, V, Cr, Si, and O. Geochimica et Cosmochimica Acta **167**, 177–194 (2015). https://doi.org/10.1016/j.gca.2015.06.026

[3] J. Badro, J. Aubert, K. Hirose, R. Nomura, I. Blanchard, S. Borensztajn, J. Siebert, Magnesium partitioning between Earth's mantle and core and its potential to drive an early exsolution geodynamo. Geophysical Research Letters **45**, 13,240–13,248 (2018). https://doi.org/10.1029/2018GL080405. URL https://onlinelibrary.wiley.com/doi/abs/10.1029/2018GL080405?af=R

[4] D. Huang, J. Badro, J. Siebert, The niobium and tantalum concentration in the mantle constrains the composition of earth's primordial magma ocean. Proceedings of the National Academy of Sciences **117**(45), 27893–27898 (2020). https://doi.org/10.1073/pnas.2007982117. URL https://www.pnas.org/content/117/45/27893

[5] D. Huang, J. Siebert, J. Badro, High pressure partitioning behavior of mo and w and late sulfur delivery during earth's core formation. Geochimica et Cosmochimica Acta **310**, 19–31 (2021). https://doi.org/https://doi.org/10.1016/j.gca.2021.06.031. URL https://www.sciencedirect.com/science/article/pii/S0016703721003896

[6] E.S. Jennings, Using complementary microanalytical techniques to analyse diamond anvil cell experiments. IOP Conference Series: Materials Science and Engineering **891**(1), 012015 (2020). https://doi.org/10.1088/1757-899X/891/1/012015. URL https://dx.doi.org/10.1088/1757-899X/891/1/012015

[7] T.A. Suer, J. Siebert, L. Remusat, J.M.D. Day, S. Borensztajn, B. Doisneau, G. Fiquet, Reconciling metal–silicate partitioning and late accretion in the Earth. Nature Communications **12**(1), 2913 (2021). https://doi.org/10.1038/s41467-021-23137-5. URL http://dx.doi.org/10.1038/s41467-021-23137-5https://www.nature.com/articles/s41467-021-23137-5

[8] I. Blanchard, D. Rubie, E. Jennings, I. Franchi, X. Zhao, S. Petitgirard, N. Miyajima, S. Jacobson, A. Morbidelli, The metal–silicate partitioning of carbon during earth's accretion and its distribution in the early solar system. Earth and Planetary Science Letters **580**, 117374 (2022). https://doi.org/https://doi.org/10.1016/j.epsl.2022.117374. URL https://www.sciencedirect.com/science/article/pii/S0012821X22000103

[9] D. Huang, J. Siebert, P. Sossi, E. Kubik, G. Avice, M. Murakami, Nitrogen sequestration in the core at megabar pressure and implications for terrestrial accretion. Geochimica et Cosmochimica Acta **376**(May), 100–112 (2024). https://doi.org/10.1016/j.gca.2024.05.010. URL https://linkinghub.elsevier.com/retrieve/pii/S0016703724002321

[10] K. Thompson, D. Lawrence, D.J. Larson, J.D. Olson, T.F. Kelly, B. Gorman, In situ site-specific specimen preparation for atom probe tomography. Ultramicroscopy **107**(2-3), 131–139 (2007). https://doi.org/10.1016/j.ultramic.2006.06.008

[11] K. Hirose, Y. Fei, Subsolidus and melting phase relations of basaltic composition in the uppermostlower mantle. Geochimica et Cosmochimica Acta **66**(12), 2099–2108 (2002). https://doi.org/https://doi.org/10.1016/S0016-7037(02)00847-5. URL https://www.sciencedirect.com/science/article/pii/S0016703702008475

[12] S. Tagawa, N. Sakamoto, K. Hirose, S. Yokoo, J. Hernlund, Y. Ohishi, H. Yurimoto, Experimental evidence for hydrogen incorporation into Earth's core. Nature Communications **12**(1), 1–8 (2021). https://doi.org/10.1038/s41467-021-22035-0. URL http://dx.doi.org/10.1038/s41467-021-22035-0

[13] M. Leschik, G. Heide, G. Frischat, H. Behrens, M. Wiedenbeck, N. Wagner, K. Heide, H. Geißler, U. Reinholz, Determination of h2o and d2o contents in rhyolitic glasses. European Journal of Glass Science and Technology Part B Physics and Chemistry of Glasses **45**, 238–251 (2004)
