# Supplementary material for: Experimental quantification of hydrogen content in the Earth’s core
Source: Nat Commun. 2026 Feb 10;17:1211. doi: 10.1038/s41467-026-68821-6 (PMC12890951; doi:10.1038/s41467-026-68821-6)
Supplement: Supplementary file 2 — Description of Additional Supplementary Files [file 41467_2026_68821_MOESM2_ESM.pdf]

Description of Additional Supplementary Files

Supplementary Data 1: Summary of diamond anvil cell and atom probe experiments.

Supplementary Data 2: Decomposed elemental abundances of the recovered metal (containing 3 nanostructures) in Fig. 2.

Supplementary Data 3: Decomposed elemental abundances of the recovered metal in Fig. 3.

Supplementary Data 4: Decomposed elemental abundances of the recovered silicate in Fig. A3.

Supplementary Data 5: Decomposed elemental abundances of the recovered silicate in Fig. A4.

Supplementary Data 6: EPMA data for starting hydrous MORB glass (1σ corresponding to 1 standard deviation of multiple measurements).

Supplementary Data 7: Estimated water content in the bulk silicate Earth.
